# 518 Autologous Skin Cell Suspensions for Reconstruction of Nonthermal Soft Tissue Wounds: A Case Series

**DOI:** 10.1093/jbcr/irad045.115

**Published:** 2023-05-15

**Authors:** Paige Deville, Elle Lovick, Jeffrey Carter, Herbert Phelan

**Affiliations:** LSUHSC New Orleans Department of General Surgery, New Orleans, Louisiana; University Medical Center- New Orleans Burn Unit, New Orleans, Louisiana; University Medical Center New Orleans & LSUHSC, River Ridge, Louisiana; University Medical Center- New Orleans Burn Center, New Orleans, Louisiana; LSUHSC New Orleans Department of General Surgery, New Orleans, Louisiana; University Medical Center- New Orleans Burn Unit, New Orleans, Louisiana; University Medical Center New Orleans & LSUHSC, River Ridge, Louisiana; University Medical Center- New Orleans Burn Center, New Orleans, Louisiana; LSUHSC New Orleans Department of General Surgery, New Orleans, Louisiana; University Medical Center- New Orleans Burn Unit, New Orleans, Louisiana; University Medical Center New Orleans & LSUHSC, River Ridge, Louisiana; University Medical Center- New Orleans Burn Center, New Orleans, Louisiana; LSUHSC New Orleans Department of General Surgery, New Orleans, Louisiana; University Medical Center- New Orleans Burn Unit, New Orleans, Louisiana; University Medical Center New Orleans & LSUHSC, River Ridge, Louisiana; University Medical Center- New Orleans Burn Center, New Orleans, Louisiana

## Abstract

**Introduction:**

Based on studies showing the use of autologous skin cell suspensions (ASCS) leads to burn wound closure with significantly smaller amounts of harvested donor skin, the Food and Drug Administration approved the first ASCS for the reconstruction of thermal injuries in September, 2018. The benefit of needing less donor skin to heal a given soft tissue wound has led to off-label usage of ASCS in a variety of clinical situations, such as necrotizing soft tissue infections (NSTI), toxic epidermal necrolysis (TEN), and traumatic injuries with soft tissue loss among others. Here, we report on a single-institution case series of ASCS usage for closure of soft tissue wounds caused by nonthermal mechanisms.

**Methods:**

We performed a retrospective chart review of all patients treated with ASCS for an indication of non-thermal soft tissue wounds at our American Burn Association-verified academic burn center between April 1, 2018 to June 1, 2022. Descriptive demographic data were collected.

**Results:**

Eight patients (6 male/2 female, mean age 57 + 17 yrs, mean length of stay 46 + 30 days) were found to have undergone closure of their wounds using ASCS during the time period of the chart review. Indications were NSTI (n=2), frostbite (n=2), antiphospholipid syndrome (n=2), and TEN (n=2). ASCS was used as a sole therapy for the TEN patients while ASCS was used in combination with meshed autografting for all other patients. The mean size of wounds treated with ASCS was 2835 + 2670 cm2, and the mean ASCS take rate was 86 + 7%. No ASCS-related complications were found.

**Conclusions:**

These results bolster the off-label use of ASCS solutions for closure of nonthermal wounds while the surgical world awaits the results of an ongoing Phase III trial on this question

**Applicability of Research to Practice:**

This study seeks to contribute to the literature supporting off-label uses of ASCS in non-thermal injuries & diseases.

